# Association between Household Crowding and Violent Discipline and Neglect of Children: Analysis of Multiple Indicator Cluster Surveys in 26 Low- and Middle-Income Countries

**DOI:** 10.3390/ijerph18041685

**Published:** 2021-02-10

**Authors:** Yaqing Gao, Xiaoyi Mi, Yinping Wang, Siyu Zou, Hong Zhou

**Affiliations:** Department of Maternal and Child Health, School of Public Health/National Health Commission Key Laboratory of Reproductive Health, Peking University, Beijing 100191, China; gaoyaqing@bjmu.edu.cn (Y.G.); hblg2015mxy@163.com (X.M.); wyping201406@bjmu.edu.cn (Y.W.); zousiyu@pku.edu.cn (S.Z.)

**Keywords:** household crowding, violent discipline, child neglect, stimulation activities, child maltreatment

## Abstract

The influence of household crowding on physical and mental health has been well documented. However, research on the influence of household crowding on violent discipline and neglect of children is scarce. Therefore, we aimed to investigate whether household crowding was associated with violent discipline and neglect of children in low- and- middle-income countries (LMICs). Cross-sectional data for 280,005 and 73,030 children in 26 LMICs surveyed using the Multiple Indicator Cluster Survey were analyzed for (1) violent discipline and (2) neglect, respectively. In each country, we used logistic regression models to estimate the effects of household crowding on multiple forms of violent discipline and stimulation activities (as a proxy of the level of child neglect). Estimates were pooled using random effects meta-analyses. After adjusting for confounding variables, household crowding was associated with higher odds of any violent discipline (odds ratio (OR) = 1.09, 95% CI 1.03 to 1.15, *p* = 0.002) and lower odds of engaging in four or more stimulation activities (OR = 0.88, 95% CI 0.83 to 0.94, *p* < 0.001). The associations were stronger for urban children and children living in low- and lower-middle-income countries. The findings suggest that screenings and interventions aimed at reducing the effects of household crowding might be effective in preventing and controlling violent discipline and neglect of children in LMICs.

## 1. Introduction

Maltreatment of children and adolescents is increasingly recognized as a significant social and public health problem. Increasing attention has been given to two forms of maltreatment: (1) violent discipline and (2) neglect. Estimates in low- and middle-income countries (LMICs) indicate that 220.4 million and 230.7 million children aged 2 to 4 years were exposed to physical punishment and psychological aggression, respectively, which corresponds to a prevalence of 62.5% and 65.4%, respectively [1]. One of the forms of neglect that has been investigated extensively in LMICs is the inadequacy of home stimulation activities [2,3,4,5]. Sixty-nine percent of children aged 3 to 4 years had received adequate home stimulation, as defined by adults engaging in at least four out of six specific, basic home stimulation activities, with the lowest proportions in sub-Saharan Africa (47%) and South Asia (74.5%) [5]. Violent discipline and neglect have been linked to serious and often lifelong consequences. Victims of violent discipline and neglect in childhood are at higher risk of health problems, including depression, anxiety, suicidal behavior, and HIV infection [6]. Exposure to violent discipline and neglect in childhood is strongly associated with behavioral and social problems such as poor cognitive performance, alcohol and drug abuse, high-risk sexual behaviors, and, once they become parents, perpetration of child maltreatment [6,7,8,9].

Most research on the risk factors of violent discipline and neglect have focused on the following: (1) children’s individual factors, such as mental and intellectual status [10] as well as sexual orientation [11]; (2) caregivers’ individual factors, such as mental health and self-esteem, acceptance of domestic violence, and history of child abuse [12]; and (3) relationship factors, such as violence in the family and parental death/separation [12]. A small number of studies have focused on environmental determinants of violent discipline and neglect of children, such as high rates of community violence [13]. However, the effects of the home environment have received little attention. Current World Health Organization (WHO) guidelines for preventing violent discipline and neglect of children have primarily focused on transforming harmful social norms around child rearing and introducing positive parenting skills, mainly through parenting programs, home visits, and media campaigns [14,15]. Identifying modifiable home environmental factors that affect violent discipline and neglect of children would provide insight into the design of interventions.

Household crowding, a condition that occurs when the number of household members exceeds the capacity of the dwelling space available, is a universal marker of an adverse home environment [16]. Common measures of crowding include persons per room, persons per bedroom, and unit square footage per person [17]. Of these measures, having more than three people per bedroom has been identified by the United Nations as one of five conditions that suggest a settlement should be characterized as a slum [18]. Studies have reported a direct association between household crowding and negative health outcomes, including tuberculosis [19], diarrheal diseases [20], and rheumatic disease [21]. Household crowding has also been linked to psychological status and behavior changes, including increased risks of depression [22], intimate partner violence [23], and homicide-suicides [24]. However, the associations of household crowding with violent discipline and neglect of children have received little attention. Two small-sized sample studies showed the association of household crowding with victimization from adult aggression in adolescents [23,25]. Studies conducted in the United States and the United Kingdom showed that a higher level of crowding in the home was associated with a lower level of parental verbal stimulation and responsivity [26,27,28]. There have been few studies that examined the associations of household crowding with different forms of violent discipline and neglect, using standardized definitions and questionnaires, in a multiple-LMIC setting.

In this study, we used data collected using the Multiple Indicator Cluster Survey (MICS) from 26 LMICs to test the hypothesis that household crowding is associated with a higher likelihood of children experiencing psychological aggression, physical punishment, and severe physical punishment as well as a lower likelihood of children’s family members engaging in stimulation activities. Furthermore, household crowding might have heterogenous effects on violent discipline and stimulation activities between population sub-groups. For example, urbanization and poverty have also been recognized as stressors [29,30]. If adults living in crowded homes are city dwellers and have little wealth, they might experience the synergistic effect of two or more stressors, which might increase the risk of perpetrating violent discipline on children. To assist in the development of more precise screenings and interventions for at-risk children living in a crowded home, we conducted stratified analyses by child sex, residence, and age, country income, and WHO regions.

## 2. Materials and Methods

### 2.1. Data Source

MICSs are population-based cross-sectional household surveys carried out at approximately 5-year intervals in a range of countries, mainly LMICs. We extracted data from the most recent round (i.e., the 6th round) of MICSs that (1) included information on household crowding; (2) assessed violent discipline and neglect of children using the standard questionnaires; (3) included complete information on sample design—the cluster, stratification, and weight variables; (4) were nationally (rather than sub-nationally) representative; and (5) were publicly available on the MICS website [31] prior to January 2021. Table 1 shows the country-wise survey characteristics of the included countries.

Using similar sampling strategies and standardized questionnaires ensures that the data are comparable across countries and over time. MICSs follow a two-stage cluster random sampling procedure. First, sampling frames were constructed using the most recent national population and housing census, and clusters were selected with the use of probability proportional to size sampling. Second, about 20 to 30 households within the sampled clusters were randomly selected. MICS data were collected in standardized face-to-face interviews with respondents, based on a set of globally recommended questionnaires that were validated using several iterations of pretesting and field surveys.

### 2.2. Outcomes

#### 2.2.1. Violent Discipline

The child discipline module in the MICS is a modified version of the Parent–Child Conflict Tactics Scale, which is a valid and reliable epidemiological instrument used to assess domestic violence against children [32]. The module was applied to children aged 1 to 14 years. For each item in the module, the mother was asked whether a certain disciplinary method had been perpetrated on the target child by any member of the household during the month preceding the interview.

We selected four outcomes: psychological aggression, physical punishment, severe physical punishment, and “any violent discipline”. Psychological aggression was defined as answering “yes” to either or both of the following 2 items: (1) shouted, yelled at, or screamed at the child, or (2) called the child dumb, lazy, or another name like that. Physical punishment was defined as answering “yes” to any of the following 6 items: (1) shook the child, (2) spanked, hit, or slapped the child on the bottom with bare hand, (3) hit or slapped the child on the hand, arm, or leg, (4) hit the child on the bottom or elsewhere on the body with something such as a belt, hairbrush, stick, or other hard object, (5) hit or slapped the child on the face, head, or ears, or (6) beat the child up—that is, hit the child over and over as hard as one could. Severe physical punishment was defined as answering “yes” to the above-mentioned (5) and/or (6). Any violent discipline was defined as answering “yes” to any of the above-mentioned items.

#### 2.2.2. Child Neglect

We used the level of adults engaging in stimulation activities with the children as a proxy for the level of child neglect [4]. The measures of stimulation activities in the MICSs were derived from the Home Observation for Measurement of the Environment (HOME) scale, which has demonstrated acceptable reliability and validity in studies conducted in several developing countries [33,34]. The measures were applied to children aged 3 to 4 years. For each of the following 6 activities, mothers were asked to respond with “yes” or “no” with regard to whether any household member aged 15 or above had engaged in that activity with the target child in the past 3 days: (1) reading books or looking at pictures, (2) telling stories, (3) singing songs, (4) taking the child outside, (5) playing with the child, and (6) naming, counting, or drawing with the child. Engaging in four or more activities was used as a proxy for adequate stimulation activities (i.e., no child neglect) [5,35]. The engagement in each of the 6 activities and engaging in four or more activities were selected as outcomes.

### 2.3. Exposures

We defined household crowding based on the definition of slums developed by the United Nations as more than three de jure household members (i.e., household members that usually live in the household, excluding visitors) per bedroom [18]. The definition was aligned with recent studies that explored household crowding and its impact on child health in LMICs [20,36,37].

### 2.4. A Priori Confounding Variables

To reduce confounding bias, we included the following covariates: age (years) and sex of the child; education, age (in 5-year age categories from 15 to 49 years), and marital status (currently/formerly/never married or in union) of the mother; whether there were more than 3 children aged 1 to 17 years in the household (yes/no), residence (rural/urban), and household wealth quintile.

Mother’s education was included as a categorical variable, with different classifications across the countries. The median number of children aged 1 to 17 years in the households was 3 in the entire sample; therefore, we dichotomized the number of children in the home into 3 or less and more than 3 for the household size. The construction of the wealth index in the MICS included selecting a basket of asset indicator variables, running a principal component analysis, calculating wealth scores that are based on the first component of the principal component analysis, and assigning the score to household members. The total household population for each country was divided into quintiles based on their wealth score and arranged from poorest (wealth quintile 1) to richest (wealth quintile 5) [38].

### 2.5. Statistical Analysis

For each country, we investigated the associations of household crowding with the outcomes using multivariate logistic regressions. In all models, we controlled for the same set of a priori defined confounding variables. Based on the study design, the survey weights and the cluster and sample strata statements were considered in all models to provide point estimates and standard errors. Only children without any missing information on the household crowding, outcomes, and all confounding variables were included in the model.

We assessed the heterogeneity of the estimated associations among countries using the I^2^ index [39]. We then conducted random effects meta-analyses with the DerSimonian and Laird method to relate the country-specific effects of household crowding on the specific outcome to the pooled estimates for the 26 countries [40].

To further reveal the heterogeneity of the effect of household crowding on violent discipline and neglect of children across different populations, we conducted sub-group analyses based on the sex, residence, and age (only for violent discipline) of the children in each country and pooled the results using the above-mentioned method. We also conducted sub-group meta-analyses according to the World Bank country classifications by income level (2020–2021) and the country groupings of the WHO. As a rule, at least 3 studies should be available per sub-group.

A *p*-value of 0.05 was considered as significant, with all analyses being conducted in R version 3.6.1 (R Foundation for Statistical Computing, Vienna, Austria).

## 3. Results

### 3.1. Study Population

All 28 countries with nationally representative MICS6 datasets publicly available on the MICS website before January 2021 included information on household crowding and violent discipline and neglect of children. Two countries (Georgia and Sierra Leone) had missing values in sample design variables and were therefore excluded from the analysis (Supplementary Table S1). Data for the analysis were extracted from surveys conducted in 26 countries dating from 2017 to 2019. Complete violent discipline, covariates, and household crowding data were available for 280,005 children (violent discipline sample), and complete stimulation activities, covariates, and household crowding data were available for 73,030 children (child neglect sample) (Table 1). The median of the national mean age of the violent discipline sample across all countries was 6.9 years (Table 2), and that of the child neglect sample was 3.5 years. In the violent discipline sample, the proportion of rural residence ranged from 21.1% in Mongolia to 81.6% in Madagascar. In Algeria, Costa Rica, Lesotho, Serbia, Tonga, Turkmenistan, and Kyrgyzstan, the proportion of mothers with less than secondary education was lower than 1%. However, in Ghana and Guinea-Bissau, more than 85% of the children’s mothers did not complete secondary education. The proportion of households having more than three children aged under 18 years ranged from 4.0% in Thailand to 82.4% in Gambia. The characteristics of the child neglect sample showed similar patterns (Supplementary Table S2).

### 3.2. Characteristics of Household Crowding and Violent Discipline and Neglect of Children

#### 3.2.1. Household Crowding

In the violent discipline sample, the median prevalence of household crowding was 26.8%, ranging from 6.2% in Costa Rica to 74.0% in Madagascar. In the child neglect sample, the median prevalence of household crowding was 28.6%, ranging from 9.7% in Costa Rica to 70.5% in Madagascar (Table 3).

#### 3.2.2. Violent Discipline and Neglect of Children

The median prevalence of psychological aggression, physical punishment, severe physical punishment, and any violent discipline is as follows (Table 3 and Supplementary Table S3): psychological aggression, 72.2% (ranging from 34.7% in Costa Rica to 89.4% in Ghana); physical punishment, 63.9% (ranging from 20.1% in Serbia to 86.5% in Kiribati); severe physical punishment, 9.0% (ranging from 0.7% in Serbia to 40.6% in DR Congo); any violent discipline, 79.7% (ranging from 44.7% in Serbia to 94.7% in Ghana). In DR Congo and Kiribati, the prevalence of both psychological aggression and physical punishment was more than 80%. In Ghana, Kiribati, and Togo, more than 90% of the children experienced at least one violent disciplinary method in the month preceding the interview.

The median prevalence of the six stimulation activities is as follows (Supplementary Table S4): reading, 55.3%; telling stories, 65.0%; singing, 70.7%; taking outside, 74.8%; playing, 80.0%; counting, 66.4%. The median prevalence of engaging in four or more activities was 72.8%, ranging from 24.8% in Gambia to 98.6% in Turkmenistan (Table 3). Gambia had the lowest prevalence among all 26 countries in singing, taking outside, playing, and counting. In Serbia, the prevalence of all the six activities was more than 80%.

### 3.3. Association of Household Crowding with Violent Discipline

Table 4 shows the associations of household crowding with violent discipline as estimated by the meta-analyses. After adjusting for a priori defined confounding variables, household crowding was associated with a 9% increase in the odds of any violent discipline (odds ratio (OR) = 1.09, 95% CI 1.03 to 1.15, *p =* 0.002). Similar associations were found for psychological aggression (OR = 1.10, 95% CI 1.04 to 1.16, *p <* 0.001), physical punishment (OR = 1.11, 95% CI 1.06 to 1.15, *p <* 0.001), and severe physical punishment (OR = 1.09, 95% CI 1.01 to 1.17, *p =* 0.03).

The association of household crowding with any violent discipline remained statistically significant only for urban residences (OR = 1.14, 95% CI 1.04 to 1.25, *p* = 0.004) and children aged 5 to 14 years old (OR = 1.10, 95% CI 1.02 to 1.18, *p* = 0.01). Heterogeneity in the association of household crowding with any violent discipline was also noted in different country income groups and WHO regions, by which the association remained significant in low-income countries (OR = 1.16, 95% CI 1.01 to 1.32, *p* = 0.04), lower-middle-income countries (OR = 1.09, 95% CI 1.03 to 1.15, *p* = 0.004), the WHO African Region (OR = 1.12, 95% CI 1.02 to 1.22, *p* = 0.01), and the WHO Southeast Asia Region (OR = 1.14, 95% CI 1.05 to 1.24, *p* = 0.002).

### 3.4. Association of Household Crowding with Child Neglect

Table 5 shows the associations of household crowding with stimulation activities as estimated by the meta-analyses. After adjusting for a priori defined confounding variables, household crowding was associated with a 12% decrease in the odds of engaging in four or more activities (OR = 0.88, 95% CI 0.83 to 0.94, *p <* 0.001). Similar associations were found for all stimulation activities except for playing with the child (OR = 0.95, 95% CI 0.89 to 1.01, *p =* 0.09).

For four or more activities, compared with the values for the other children in the sample, stronger negative associations with household crowding were found among the children who lived in urban areas (OR = 0.82, 95% CI 0.75 to 0.89, *p* < 0.001). In the subgroup meta-analyses, the association of household crowding with engaging in four or more activities remained significant in lower-middle-income countries (OR = 0.86, 95% CI 0.80 to 0.93, *p* < 0.001) and the WHO Southeast Asia Region (OR = 0.81, 95% CI 0.73 to 0.90, *p* < 0.001).

## 4. Discussion

According to our literature review, this is the first multi-country study examining the association of household crowding with violent discipline and neglect of children. Overall, we found that household crowding was associated with 9% higher odds of any violent discipline and 12% lower odds of engaging in four or more stimulation activities. The associations were stronger for children living in urban areas and children living in low- and lower-middle-income countries.

Our findings are consistent with previous studies conducted in LMICs that investigated the effects of household crowding on domestic violent discipline towards adolescents. A self-report index of household crowding was positively associated with higher scores of violent discipline among high school students in Egypt [25]. Furthermore, a study conducted in Nigeria showed that for victimization from adult aggression, adolescents who lived in apartments with only one bedroom scored significantly higher than adolescents living in apartments with more than one bedroom [23]. We extended these associations to children aged 1 to 14 years by showing the negative effects of household crowding on violent discipline, both psychological aggression and physical punishment, and the effects being stronger for children aged 5 to 14 years than for children under 5 years of age.

The association of household crowding and parental stimulation has been documented only in developed countries. A study conducted in the United States showed that the household crowding index, assessed by persons per room, was associated with parents being less verbally responsive and speaking in less sophisticated ways with their children [27]. Other studies conducted in the United States and the United Kingdom showed that the value of persons per room was associated with less maternal responsiveness, assessed by the HOME Inventory, which consists of items including spontaneously praising the child, conversing freely with the child, and caring and kissing the child [28]. We increased the generalizability of this finding by extending it to multiple LMICs.

Several mechanisms might explain the association of household crowding with violent discipline and neglect of children. Household crowding has been identified as an unmanageable and chronic stressor [41]. Managing such stressors might lead to an overload of available coping resources, threatening regulation of social interaction [42] and generating various behavioral and psychological health problems. First, frustration and depression may arise in response to the stress resulting from household crowding [22]. Poor mental health, in turn, is associated with both inadequate stimulation and harsh discipline toward one’s children [43]. Second, household crowding might stimulate aggression in individuals, which increases the risk of children facing victimization by adults. Studies have shown the negative effects of household crowding on self-reported levels of aggression and intimate partner violence in adults [23,42]. Third, individuals living in crowded environments might try to withdraw and separate themselves from others as a way of coping with stress that diminishes excessive, unwanted social interaction [42]. Social withdrawal within the home might result in unresponsive parenting [28], and social withdrawal outside the home might disrupt or erode neighborhood friendships [44] which can act as a source of social support and have been recognized as a factor associated with reduced violent discipline [45]. Fourth, the stress of household crowding might lead to alcohol abuse [46], which is a well-known risk factor for violent discipline and neglect of children [47].

We observed that point estimations of the effects of household crowding on violent discipline and neglect of children were higher in urban and poor areas. Studies on the effects of household crowding on domestic violence between adults have shown a similar pattern, in that a significant association was found in the metropolitan area of a poorer country (Lagos, Nigeria) [23], compared to that of a nationally representative sample in a wealthier country (South Africa) [48]. Urbanization was the predictor for violent discipline [25], while poverty was the risk factor for violent discipline and inadequate stimulation [5,49]. A major negative effect that resulted from urbanization and poverty was stress [29,30], which might affect child maltreatment through synergism with household crowding. In addition, individuals living in rural areas generally spent more time outdoors [50], which could reduce the cumulative exposure of household crowding, offer respite for mental health [51], and, therefore, might reduce the potential for hostile and unresponsive behavior of adults towards children. Moreover, we observed that children aged 5 to 14 years were more susceptible to the effects of household crowding on violent discipline than younger children. Studies have indicated that the peak age range for experiencing non-fatal physical abuse was 6 to 11 in India [52]. Therefore, the child’s age, in combination with household crowding, could synergistically affect the likelihood of violent discipline.

This study had several limitations. First, we were unable to establish a causal relationship between household crowding and violent discipline and neglect of children due to the cross-sectional nature of the data. Second, although we hypothesized several mechanisms, the MICSs did not provide sufficiently detailed measures on several psychological mediators or causal designs to conduct a direct mediation analysis. The elucidation of potential mechanisms could be an important next step in this field of research. Third, there is currently no scientific consensus on the best way to measure household crowding. Studies conducted in developed countries usually adopt more stringent definitions, such as more than one person per bedroom, or more detailed definitions considering the occupants’ age, sex, and relationship. We defined household crowding as more than three people per bedroom, which aligns with the United Nations definition and studies conducted in LMICs [20,36,37]; this dichotomous definition is an explicit marker that facilitates screening for at-risk children. However, the prevalence of household crowding and the extent of its impact on violent discipline and neglect of children are not comparable to other studies that have adopted different definitions. Fourth, although the measures of violent discipline and neglect of children in the MICSs have shown validity in field studies [32,33,34], adults might underreport violent discipline due to fear of retribution [53]. The frequency of different modes of violent discipline varied greatly in our study. Therefore, when we used the combined variable “any violent discipline” as an outcome, the point estimations might have been biased toward that of the most frequent modes. Fifth, our measurements were less able to collect information on the chronicity and severity of the violent discipline or the duration, frequency, and quality of the stimulation activities compared with direct observation. Such non-differential misclassification generally biases the effect estimates downwards [54], which might explain the non-significant association between household crowding and playing with the child in our study.

The study’s strength is that we used a large, multi-national representative sample, rather than restricting the study to one country or region, in order to enhance the external validity of these findings. This is especially important given the cultural differences in child maltreatment disclosure and the subjective acceptability of crowdedness across regions and countries. Furthermore, we combined the results using meta-analyses to minimize bias in estimations by allowing the effect of confounding variables to be different in each country, which cannot be achieved with a one-stage analysis [55]. The use of national survey data in meta-analyses minimizes the impact of selection and publication bias. Moreover, according to Vygotsky’s cultural-historical theory of child development [56], interrelations between children and their surrounding environment is a vital source for the development of higher mental functions. There are various aspects of environment that are significant for a child, such as physical conditions, caregiving practice, and culturally and/or historically formed social norms and values. This is one of the few studies to explore the associations of different aspects of the environment and how they vary according to other environmental aspects (i.e., urbanicity, wealth, and geographical region), which could lay a foundation to further clarify the role of environment on child development.

Housing interventions have received growing attention as a long-term, sustainable, and multi-sectoral intervention package anchored in physical and mental health [16]. Low-income women who relocated to newly constructed homes reported significant improvements in mental health as a result of decreased crowding [57]. Urgent research is needed to determine whether the ongoing slum upgrading, especially the relieving of household crowding, in LMICs is preventative against violent discipline and neglect of children. Moreover, given the present level of evidence, as well as the financial factors, it would be difficult to scale actual rebuilding or relocating interventions for housing in LMICs. Therefore, targeted screening of children living in crowded houses might be effective in early identification of children at risk of violent victimization and connecting them with essential child protection services and stimulation activities. Targeting populations that are more susceptible to the effects of household crowding on violent discipline and neglect of children might maximize the cost-effectiveness of the interventions.

## 5. Conclusions

This constitutes the first multi-country study of LMICs to demonstrate the associations of household crowding with violent discipline and neglect of children. Specifically, our findings highlight that the associations were most apparent in urban and poor children. Given the cross-sectional nature of the data, this study is inherently exploratory. Future work to establish a causal link between household crowding and violent discipline and neglect of children, to assess its underlying mechanisms, and to explore more environmental determinants of the outcomes is crucial for designing interventions and screenings for child maltreatment in LMICs.

## Figures and Tables

**Table 1:** Survey characteristics of the 26 countries included in the analysis.

Country	Survey Year	Country Income	WHO Region	Violent Discipline Sample (N) ^a^	Child Neglect Sample (N) ^b^
Algeria	2018–2019	Lower-middle	AFR	23,057	5785
Bangladesh	2019	Lower-middle	SEAR	45,991	9255
DR Congo	2017–2018	Low	AFR	24,784	7755
Costa Rica	2018	Upper-middle	AMR	5747	1482
Gambia	2018	Low	AFR	11,314	3875
Ghana	2017–2018	Lower-middle	AFR	11,689	3208
Guinea-Bissau	2018–2019	Low	AFR	8790	2698
Iraq	2018	Upper-middle	EMR	24,505	6939
Kiribati	2018–2019	Lower-middle	WPR	3126	779
Kosovo	2019–2020	Upper-middle	EUR	2805	607
Kyrgyzstan	2018	Lower-middle	EUR	5191	1327
Lao	2017	Lower-middle	WPR	19,032	4501
Lesotho	2018	Lower-middle	AFR	4293	929
Madagascar	2018	Low	AFR	14,372	4593
Mongolia	2018	Lower-middle	WPR	5323	1307
Montenegro	2018	Upper-middle	EUR	1704	481
Nepal	2019	Lower-middle	SEAR	10,909	2801
North Macedonia	2018–2019	Upper-middle	EUR	2329	641
Serbia	2019	Upper-middle	EUR	2837	744
Suriname	2018	Upper-middle	AMR	5461	1531
Thailand	2019	Upper-middle	SEAR	17,695	4319
Togo	2017	Low	AFR	6596	1783
Tonga	2019	Upper-middle	WPR	1908	520
Tunisia	2018	Lower-middle	EMR	6136	1477
Turkmenistan	2019	Upper-middle	EUR	5847	1554
Zimbabwe	2019	Lower-middle	AFR	8564	2139

AFR, African Region; AMR, Region of the Americas; EUR, European Region; EMR, Eastern Mediterranean Region; SEAR, Southeast Asia Region; WHO, World Health Organization; WPR, Western Pacific Region. ^a^ Number of children aged 1 to 14 years without missing information on violent discipline, confounding variables, and household crowding. ^b^ Number of children aged 3 to 4 years without missing information on stimulation activities, confounding variables, and household crowding.

**Table 2:** Demographic characteristics of the violent discipline sample.

Country	Total Sample (N) ^a^	Child’s Mean Age (Years) ^b^	Female (%)	Rural (%)	Education (%) ^c^	Mean Age (Years) ^d^	Marital Status (%) ^e^	Number of Children (%) ^f^	Household Wealth (%) ^g^
Algeria	23,057	6.9	48.5	39.2	0.0	37.0	96.7	34.8	44.4
Bangladesh	45,991	7.5	49.3	79.3	46.5	32.2	97.2	15.3	43.1
DR Congo	24,784	6.3	50.2	59.0	54.6	33.3	86.8	69.6	42.9
Costa Rica	5747	7.1	47.8	32.3	0.0	33.1	70.0	8.0	46.6
Gambia	11,314	6.7	52.1	36.3	72.4	33.7	94.0	82.4	43.8
Ghana	11,689	6.9	49.4	56.6	88.1	35.2	86.3	57.3	43.7
Guinea-Bissau	8790	6.6	50.1	68.7	91.6	33.2	86.6	70.5	41.8
Iraq	24,505	7.2	49.0	32.1	66.9	34.1	97.0	66.3	45.0
Kiribati	3126	6.9	49.5	48.2	19.9	34.5	92.5	52.0	42.4
Kosovo	2805	7.7	48.1	59.1	17.1	35.9	98.4	31.3	47.5
Kyrgyzstan	5191	6.9	47.4	66.9	0.4	34.0	94.7	38.3	44.8
Lao	19,032	7.3	49.2	74.8	69.8	33.1	96.2	33.8	49.8
Lesotho	4293	7.1	51.3	62.2	0.0	33.3	77.0	26.2	41.0
Madagascar	14,372	6.0	49.8	81.6	78.7	31.6	85.4	56.7	51.4
Mongolia	5323	6.7	48.2	21.1	5.3	34.7	90.5	17.7	16.7
Montenegro	1704	6.7	49.5	33.2	71.0	34.9	95.3	20.3	38.9
Nepal	10,909	7.1	48.1	34.7	69.2	31.8	98.1	23.6	42.6
North Macedonia	2329	6.3	49.0	39.5	30.6	34.3	95.8	11.5	44.1
Serbia	2837	6.4	46.3	39.1	0.0	35.1	92.4	7.0	31.7
Suriname	5461	6.5	48.5	32.4	27.5	33.9	84.3	39.8	51.0
Thailand	17,695	6.5	49.4	59.9	24.4	35.3	89.7	4.0	36.1
Togo	6596	6.9	48.1	61.6	77.7	34.4	92.1	10.2	43.8
Tonga	1908	7.2	47.7	79.1	0.0	36.2	91.8	57.8	46.0
Tunisia	6136	7.2	48.4	33.0	40.8	37.4	97.0	19.0	39.9
Turkmenistan	5847	6.8	48.2	59.2	0.0	33.7	94.5	37.7	42.5
Zimbabwe	8564	6.8	49.9	70.6	34.3	33.8	84.1	40.2	45.6
Total ^h^	280,005	6.9	49.0	57.8	32.5	34.0	92.4	34.3	43.7

^a^ Number of children aged 1 to 14 years without missing information on violent discipline, confounding variables, and household crowding. ^b^ Estimates of mean or percentage of the characteristics considering the sample weight and the cluster and sample strata statements, based on the country-specific study design. ^c^ Estimates are the percentage of children whose mother had less than secondary education. ^d^ Estimates are the mean for mother’s age. ^e^ Estimates are the percentage of children whose mother was married/in union at the time of the interview. ^f^ Estimates are the percentage of households that had more than three children aged under 18 years at home. ^g^ Estimates are the percentage of households that belonged to the bottom two wealth quintiles. ^h^ Estimates are the median of the mean or percentage of the characteristics of all countries.

**Table 3:** Characteristics of household crowding, violent discipline, and stimulation activities of study participants.

Country	Violent Discipline Sample		Child Neglect Sample	
	Household Crowding (%) ^a^	Any Violent Discipline (%)	Household Crowding (%)	Four or More Activities (%)
Algeria	26.4	85.2	31.1	73.1
Bangladesh	27.3	89.5	28.4	70.4
DR Congo	41.8	89.6	42.2	58.2
Costa Rica	6.2	50.3	9.7	84.7
Gambia	22.7	89.7	23.3	24.8
Ghana	50.8	94.7	53.5	44.3
Guinea-Bissau	20.8	76.6	19.9	55.7
Iraq	49.4	81.5	51.3	61.2
Kiribati	57.5	92.3	57.5	82.8
Kosovo	20.7	71.7	25.1	78.2
Kyrgyzstan	21.6	75.0	24.9	91.5
Lao	52.8	69.5	54.1	56.7
Lesotho	45.4	77.9	45.5	36.9
Madagascar	74.0	87.5	70.5	34.8
Mongolia	44.4	51.0	48.0	72.6
Montenegro	24.9	66.9	27.4	96.2
Nepal	27.1	82.3	28.8	83.8
North Macedonia	15.0	73.3	16.3	95.1
Serbia	10.0	44.7	15.5	98.3
Suriname	28.3	88.8	29.1	72.3
Thailand	17.5	57.6	18.9	97.4
Togo	35.1	92.2	38.1	41.8
Tonga	23.9	86.7	24.5	91.9
Tunisia	16.7	88.8	19.6	84.7
Turkmenistan	14.2	69.5	17.8	98.6
Zimbabwe	27.2	65.8	30.5	47.0
Total ^b^	26.8	79.7	28.6	72.8

^a^ Estimates of percentages of the characteristics considering the sample weight and the cluster and sample strata statements, based on the country-specific study design. ^b^ Estimates are the median of the percentage of the characteristics of all countries.

**Table 4:** Association of household crowding with violent discipline in children aged 1 to 14 years.

Country	OR (95% CI)	*p* Value	I^2^ (%)
Association of household crowding with different types of violent discipline			
Psychological aggression	1.10 (1.04 to 1.16)	<0.001	46.3
Physical punishment	1.11 (1.06 to 1.15)	<0.001	25.4
Severe physical punishment	1.09 (1.01 to 1.17)	0.03	57.0
Any violent discipline	1.09 (1.03 to 1.15)	0.002	33.4
Stratified associations of household crowding with any violent discipline			
Child sex			
Female	1.09 (1.02 to 1.17)	0.01	31.0
Male	1.08 (1.00 to 1.16)	0.04	30.2
Residence			
Rural	1.05 (0.97 to 1.12)	0.21	36.7
Urban	1.14 (1.04 to 1.25)	0.004	40.2
Child age (years)			
1–4	1.03 (0.98 to 1.08)	0.28	7.2
5–14	1.10 (1.02 to 1.18)	0.01	31.3
Country income			
Low-income	1.16 (1.01 to 1.32)	0.04	37.1
Lower-middle income	1.09 (1.03 to 1.15)	0.004	22.4
Upper-middle income	1.05 (0.91 to 1.21)	0.49	45.4
WHO region			
AFR	1.12 (1.02 to 1.22)	0.01	38.8
EUR	1.10 (0.89 to 1.36)	0.38	56.5
SEAR	1.14 (1.05 to 1.24)	0.002	0.0
WPR	1.07 (0.98 to 1.17)	0.11	0.0

OR, odds ratio; CI, confidence interval; AFR, African Region; EUR, European Region; SEAR, Southeast Asia Region; WHO, World Health Organization; WPR, Western Pacific Region.

**Table 5:** Association of household crowding with child neglect in children aged 3 to 4 years.

Country	OR (95% CI)	*p* Value	I^2^ (%)
Association of household crowding with different types of stimulation activities			
Reading	0.85 (0.80 to 0.92)	<0.001	28.3
Telling stories	0.87 (0.81 to 0.92)	<0.001	25.4
Singing	0.86 (0.82 to 0.90)	<0.001	0.0
Taking outside	0.88 (0.82 to 0.94)	<0.001	18.6
Playing	0.95 (0.89 to 1.01)	0.09	17.1
Counting	0.91 (0.85 to 0.97)	0.005	29.3
Four or more activities	0.88 (0.83 to 0.94)	<0.001	8.1
Stratified associations of household crowding with engaging in four or more stimulation activities			
Child sex			
Female	0.86 (0.79 to 0.95)	0.002	23.1
Male	0.89 (0.83 to 0.96)	0.003	2.5
Residence			
Rural	0.92 (0.85 to 1.00)	0.05	19.9
Urban	0.82 (0.75 to 0.89)	<0.001	2.9
Country income			
Low-income	0.96 (0.86 to 1.06)	0.41	0.0
Lower-middle income	0.86 (0.80 to 0.93)	<0.001	16.2
Upper-middle income	0.85 (0.72 to 1.01)	0.06	0.0
WHO region			
AFR	0.93 (0.86 to 1.01)	0.09	0.0
EUR	0.89 (0.63 to 1.25)	0.49	0.0
SEAR	0.81 (0.73 to 0.90)	<0.001	0.0
WPR	0.95 (0.70 to 1.30)	0.75	64.3

OR, odds ratio; CI, confidence interval; AFR, African Region; EUR, European Region; SEAR, Southeast Asia Region; WHO, World Health Organization; WPR, Western Pacific Region.

## Data Availability

The data presented in this study are openly available at http://mics.unicef.org/surveys.

## Supplementary Materials

The following are available online at https://www.mdpi.com/1660-4601/18/4/1685/s1, Table S1: Selection of countries; Table S2: Demographic characteristics of the child neglect sample; Table S3: Characteristics of different types of violent discipline; Table S4: Characteristics of different types of stimulation activities; Table S5: Association of household crowding with any violent discipline in 26 countries; Table S6: Association of household crowding with engaging in four or more stimulation activities in 26 countries.
