# Supplementary material for: Association between Household Crowding and Violent Discipline and Neglect of Children: Analysis of Multiple Indicator Cluster Surveys in 26 Low- and Middle-Income Countries
Source: Int J Environ Res Public Health. 2021 Feb 10;18(4):1685. doi: 10.3390/ijerph18041685 (PMC7916333; doi:10.3390/ijerph18041685)
Supplement: Supplementary file 1 [file ijerph-18-01685-s001.pdf]

Table S1. Selection of countries.

Country [a]	Survey Year	Country in-Come	WHO Region	Violent Discipline Sample (N) [b]	Child Neglect Sample (N) [c]	Included in the Analysis	Reason for Exclusion
Algeria	2018-2019	Lower-middle	AFR	23057	5785	Yes	-
Bangladesh	2019	Lower-middle	SEAR	45991	9255	Yes	-
DR Congo	2017-2018	Low	AFR	24784	7755	Yes	-
Costa Rica	2018	Upper-middle	AMR	5747	1482	Yes	-
Gambia	2018	Low	AFR	11314	3875	Yes	-
Georgia	2018	Upper-middle	EUR	4606	1064	No	Missing values in the weight variable
Ghana	2017-2018	Lower-middle	AFR	11689	3208	Yes	-
Guinea-Bissau	2018-2019	Low	AFR	8790	2698	Yes	-
Iraq	2018	Upper-middle	EMR	24505	6939	Yes	-
Kiribati	2018-2019	Lower-middle	WPR	3126	779	Yes	-
Kosovo	2019-2020	Upper-middle	EUR	2805	607	Yes	-
Kyrgyzstan	2018	Lower-middle	EUR	5191	1327	Yes	-
Lao	2017	Lower-middle	WPR	19032	4501	Yes	-
Lesotho	2018	Lower-middle	AFR	4293	929	Yes	-
Madagascar	2018	Low	AFR	14372	4593	Yes	-
Mongolia	2018	Lower-middle	WPR	5323	1307	Yes	-
Montenegro	2018	Upper-middle	EUR	1704	481	Yes	-
Nepal	2019	Lower-middle	SEAR	10909	2801	Yes	-
North Macedonia	2018-2019	Upper-middle	EUR	2329	641	Yes	-
Serbia	2019	Upper-middle	EUR	2837	744	Yes	-
Sierra Leone	2017	Low	AFR	14719	4012	No	Missing values in the stratification variable
Suriname	2018	Upper-middle	AMR	5461	1531	Yes	-
Thailand	2019	Upper-middle	SEAR	17695	4319	Yes	-
Togo	2017	Low	AFR	6596	1783	Yes	-
Tonga	2019	Upper-middle	WPR	1908	520	Yes	-
Tunisia	2018	Lower-middle	EMR	6136	1477	Yes	-
Turkmenistan	2019	Upper-middle	EUR	5847	1554	Yes	-
Zimbabwe	2019	Lower-middle	AFR	8564	2139	Yes	-

AFR, African Region; AMR, Region of the Americas; EUR, European Region; EMR, Eastern Mediterranean Region; SEAR, Southeast Asia Region; WHO, World Health Organization; WPR, Western Pacific Region. [a] Countries with nationally representative 6th round of MICS datasets publicly available on the MICS website (http://mics.unicef.org/surveys) prior to January 2021. [b] Number of children aged 1 to 14 years without missing information on violent discipline, confounding variables, and household crowding. [c] Number of children aged 3 to 4 years without missing information on stimulation activities, confounding variables, and household crowding.

Table S2. Demographic characteristics of the child neglect sample.

Country	Total Sample (N) [a]	Female (%) [b]	Rural (%)	Education (%) [c]	Mean Age (years) [d]	Marital Status (%) [e]	Number of Children (%) [f]	Household Wealth (%) [g]
Algeria	5785	48.5	41.1	0.0	34.1	97.8	28.7	48.5
Bangladesh	9255	48.3	79.0	36.6	28.6	98.2	13.7	48.3
DR Congo	7755	50.6	61.2	54.5	31.6	87.2	63.9	50.6
Costa Rica	1482	47.6	34.3	0.0	29.9	69.9	12.3	47.6
Gambia	3875	48.0	38.5	70.8	31.5	93.9	81.1	48.0
Ghana	3208	51.7	56.8	86.2	33.0	85.9	54.3	51.7
Guinea-Bissau	2698	48.0	73.2	91.9	31.2	87.4	68.3	48.0
Iraq	6939	48.4	32.6	64.9	31.1	98.1	59.8	48.4
Kiribati	779	49.6	47.5	21.1	31.9	91.6	48.5	49.6
Kosovo	607	49.5	59.7	38.7	32.3	99.2	28.7	49.5
Kyrgyzstan	1327	50.3	67.9	0.4	31.0	95.8	33.6	50.3
Lao	4501	49.0	72.0	63.8	29.9	96.3	29.6	49.0
Lesotho	929	50.0	59.8	0.0	30.9	77.1	23.2	50.0
Madagascar	4593	49.1	79.3	73.8	30.3	83.7	44.7	49.1
Mongolia	1307	47.6	20.3	3.9	32.0	91.9	15.0	47.6
Montenegro	481	44.3	33.5	61.9	33.1	96.3	17.5	44.3
Nepal	2801	48.1	34.3	61.2	28.4	99.0	22.5	48.1
North Macedonia	641	44.4	36.8	24.1	31.7	97.8	7.9	44.4
Serbia	744	48.0	40.0	0.0	32.3	94.0	6.9	48.0
Suriname	1531	49.7	35.9	27.3	31.4	85.5	37.3	49.7
Thailand	4319	49.8	60.7	21.0	33.0	89.2	5.2	49.8
Togo	1783	47.3	61.1	73.6	31.7	92.9	15.7	47.3
Tonga	520	46.4	79.9	0.0	32.9	89.4	53.4	46.4
Tunisia	1477	50.3	34.5	32.9	34.3	97.9	15.4	50.3
Turkmenistan	1554	51.0	62.4	0.0	30.6	95.6	34.7	51.0
Zimbabwe	2139	50.6	69.6	32.6	31.8	85.6	33.6	50.6
Total [h]	73030	48.7	58.3	32.7	31.6	93.4	29.2	48.7

[a] Number of children aged 3 to 4 years without missing information on stimulation activities, confounding variables, and household crowding. [b] Estimates of mean or percentage of the characteristics considering the sample weight and the cluster and sample strata statements, based on the country-specific study design. [c] Estimates are the percentage of children whose mother had less than secondary education. [d] Estimates are the mean for mother's age. [e] Estimates are the percentage of children whose mother was married/in union at the time of the interview. [f] Estimates are the percentage of households that had more than 3 children aged under 18 years at home. [g] Estimates are the percentage of households that belonged to the bottom 2 wealth quintiles. [h] Estimates are the median of the mean or percentage of the characteristics of all countries.

Table S3. Characteristics of different types of violent discipline.

Country	Total Sample (N) [a]	Psychological Aggression (%) [b]	Physical Punishment (%)	Severe Physical Punishment (%)
Algeria	23057	78.7	68.5	17.5
Bangladesh	45991	86.9	66.0	30.9
DR Congo	24784	80.7	82.1	40.6
Costa Rica	5747	34.7	34.3	2.7
Gambia	11314	81.1	72.9	16.2
Ghana	11689	89.4	79.0	17.9
Guinea-Bissau	8790	47.4	70.7	20.2
Iraq	24505	79.1	59.6	31.5
Kiribati	3126	81.3	86.5	24.6
Kosovo	2805	68.8	30.2	5.7
Kyrgyzstan	5191	70.5	49.4	5.6
Lao	19032	66.2	34.8	4.8
Lesotho	4293	59.0	63.7	7.7
Madagascar	14372	80.8	67.9	9.3
Mongolia	5323	42.3	29.5	5.2
Montenegro	1704	63.8	32.4	4.4
Nepal	10909	77.3	64.1	20.5
North Macedonia	2329	67.1	45.5	7.0
Serbia	2837	40.1	20.1	0.7
Suriname	5461	85.2	65.6	8.7
Thailand	17695	38.9	45.0	2.1
Togo	6596	86.3	77.2	21.2
Tonga	1908	73.9	80.0	23.3
Tunisia	6136	84.8	72.8	22.7
Turkmenistan	5847	58.8	50.0	0.9
Zimbabwe	8564	55.4	43.9	6.7
Total [c]	280005	72.2	63.9	9.0

[a] Number of children aged 1 to 14 years without missing information on violent discipline, confounding variables, and household crowding. [b] Estimates of mean or percentage of the characteristics considering the sample weight and the cluster and sample strata statements, based on the country-specific study design. [c] Estimates are the median of the percentage of the characteristics of all countries.

Table S4. Characteristics of different types of stimulation activities.

Country	Total Sample (N) [a]	Reading (%) [b]	Telling stories (%)	Singing (%)	Taking Outside (%)	Playing (%)	Counting (%)
Algeria	5785	47.8	66.1	64.5	83.7	80.4	61.6
Bangladesh	9255	70.5	70.1	60.5	73.2	62.0	63.2
DR Congo	7755	19.1	53.5	68.9	65.1	71.3	34.0
Costa Rica	1482	68.1	65.4	82.5	84.8	91.7	69.7
Gambia	3875	12.6	25.7	36.4	45.1	48.9	15.6
Ghana	3208	31.1	31.2	51.6	55.0	72.1	36.5
Guinea-Bissau	2698	30.0	54.8	65.5	58.6	74.5	38.9
Iraq	6939	31.2	44.8	53.0	60.8	79.6	53.7
Kiribati	779	64.0	80.7	86.1	75.2	87.8	69.7
Kosovo	607	46.3	64.6	60.6	83.8	84.8	59.4
Kyrgyzstan	1327	83.0	88.6	77.5	92.7	92.2	82.9
Lao	4501	42.9	37.6	46.3	65.7	65.5	51.7
Lesotho	929	27.0	36.4	46.0	48.0	59.0	25.3
Madagascar	4593	12.4	17.6	37.9	64.1	52.2	38.6
Mongolia	1307	58.7	55.5	73.3	64.7	77.1	71.6
Montenegro	481	83.8	93.0	87.7	94.8	99.3	82.0
Nepal	2801	73.8	68.7	75.7	87.2	90.1	73.0
North Macedonia	641	71.7	88.9	85.6	93.8	98.9	83.3
Serbia	744	87.7	91.4	86.8	99.2	99.0	85.5
Suriname	1531	51.9	62.1	72.6	74.3	74.9	72.5
Thailand	4319	89.5	81.9	81.7	97.3	97.9	95.9
Togo	1783	16.9	33.0	46.4	59.9	64.8	21.8
Tonga	520	79.4	86.4	89.0	91.8	94.7	76.0
Tunisia	1477	61.8	66.5	76.0	75.5	92.5	82.0
Turkmenistan	1554	75.1	89.0	82.4	96.1	99.4	70.8
Zimbabwe	2139	23.4	45.7	55.4	66.9	63.8	39.4
Total [c]	73030	55.3	65.0	70.7	74.8	80.0	66.4

[a] Number of children aged 1 to 14 years without missing information on stimulation activities, confounding variables, and household crowding. [b] Estimates of mean or percentage of the characteristics considering the sample weight and the cluster and sample strata statements, based on the country-specific study design. [c] Estimates are the median of the percentage of the characteristics of all countries.

Table S5. Association of household crowding with any violent discipline in 26 countries.

Country	OR (95% CI)	*p* Value
Algeria	1.18 (1.02 to 1.35)	0.02
Bangladesh	1.16 (1.05 to 1.28)	0.005
DR Congo	1.30 (1.09 to 1.55)	0.004
Costa Rica	1.21 (0.86 to 1.71)	0.27
Gambia	1.34 (1.00 to 1.79)	0.05
Ghana	1.19 (0.92 to 1.54)	0.18
Guinea-Bissau	1.05 (0.82 to 1.35)	0.68
Iraq	0.84 (0.67 to 1.06)	0.14
Kiribati	1.16 (0.80 to 1.68)	0.42
Kosovo	0.76 (0.56 to 1.02)	0.07
Kyrgyzstan	1.28 (0.98 to 1.68)	0.08
Lao	1.11 (1.00 to 1.23)	0.06
Lesotho	0.86 (0.70 to 1.08)	0.19
Madagascar	0.95 (0.76 to 1.18)	0.63
Mongolia	0.97 (0.80 to 1.17)	0.74
Montenegro	1.61 (0.93 to 2.79)	0.09
Nepal	1.04 (0.87 to 1.25)	0.63
North Macedonia	0.97 (0.65 to 1.43)	0.88
Serbia	0.98 (0.64 to 1.48)	0.91
Suriname	1.06 (0.76 to 1.47)	0.74
Thailand	1.23 (0.98 to 1.54)	0.07
Togo	1.22 (0.90 to 1.66)	0.21
Tonga	0.91 (0.51 to 1.60)	0.73
Tunisia	0.88 (0.68 to 1.15)	0.36
Turkmenistan	1.31 (1.02 to 1.69)	0.03
Zimbabwe	1.09 (0.95 to 1.25)	0.22

OR, odds ratio; CI, confidence interval.

Table S6. Association of household crowding with engaging in four or more stimulation activities in 26 countries.

Country	OR (95% CI)	*p* Value
Algeria	0.92 (0.77 to 1.10)	0.38
Bangladesh	0.82 (0.73 to 0.92)	<0.001
DR Congo	1.03 (0.84 to 1.25)	0.79
Costa Rica	0.51 (0.30 to 0.87)	0.01
Gambia	0.88 (0.66 to 1.17)	0.37
Ghana	0.94 (0.75 to 1.19)	0.62
Guinea-Bissau	0.94 (0.70 to 1.26)	0.67
Iraq	0.91 (0.72 to 1.15)	0.41
Kiribati	0.88 (0.56 to 1.38)	0.57
Kosovo	0.91 (0.55 to 1.51)	0.71
Kyrgyzstan	0.67 (0.38 to 1.20)	0.18
Lao	0.77 (0.66 to 0.90)	0.001
Lesotho	1.02 (0.68 to 1.53)	0.94
Madagascar	0.88 (0.73 to 1.06)	0.18
Mongolia	1.31 (0.95 to 1.80)	0.10
Montenegro	0.41 (0.04 to 3.88)	0.43
Nepal	0.82 (0.62 to 1.08)	0.15
North Macedonia	1.64 (0.56 to 4.75)	0.36
Serbia	1.17 (0.35 to 3.87)	0.80
Suriname	0.90 (0.60 to 1.35)	0.60
Thailand	0.58 (0.28 to 1.20)	0.15
Togo	1.18 (0.85 to 1.66)	0.33
Tonga	1.03 (0.36 to 2.89)	0.96
Tunisia	0.90 (0.61 to 1.34)	0.61
Turkmenistan	- [a]	-
Zimbabwe	0.82 (0.65 to 1.04)	0.10

OR, odds ratio; CI, confidence interval. [a] Model did not converge.
